# Development of an HPLC Method for Absolute Quantification and QAMS of Flavonoids Components in *Psoralea corylifolia* L.

**DOI:** 10.1155/2015/792637

**Published:** 2015-10-26

**Authors:** Cuiping Yan, Yu Wu, Zebin Weng, Qianqian Gao, Guangming Yang, Zhipeng Chen, Baochang Cai, Weidong Li

**Affiliations:** ^1^Jiangsu Key Laboratory of Chinese Medicine Processing, Engineering Center of State Ministry of Education for Standardization of Chinese Medicine Processing, Nanjing University of Chinese Medicine, Nanjing 210023, China; ^2^Pharmacy College of Nanjing University of TCM, Nanjing 210023, China

## Abstract

The seeds of *Psoralea corylifolia* L. (Fabaceae) are a commonly used medicinal herb in eastern Asia with many beneficial effects in clinical therapies. In this study, a simple, sensitive, precise, and specific reverse phase high-performance liquid chromatography (HPLC) method was established for quantification of 9 flavonoids in *P. corylifolia*, including isobavachin, neobavaisoflavone, bavachin, corylin, bavachalcone, bavachinin, isobavachalcone, corylifol A, and 4′-O-methylbavachalcone. Based on this method, a quantitative analysis of multicomponents by single marker (QAMS) was carried out, and the relative correction factors (RCFs) were calculated for determining the contents of other flavonoids. The accuracy of QAMS method was verified by comparing with the results of external standard method, as well as the feasibility and adaptability of the method applied on quality control of *P. corylifolia*. The 9 compounds were baseline separated in 60 min with a good linearity of regression coefficient (*R* ^2^) over 0.9991. The accuracies of QAMS were between 92.89% and 109.5%. The RSD values of *f* in different injection volume were between 2.3% and 3.6%. The results obtained from QAMS suggested that it was a convenient and accurate method to determine multicomponents especially when some authentic standard substances were unavailable. It can be used to control the quality of *P. corylifolia*.

## 1. Introduction

*Psoralea corylifolia* L. (Fabaceae) is one of the most popular traditional Chinese medicines and officially listed in Chinese Pharmacopoeia, which is widely used for the treatment of coronary artery disease, osteoporosis, bacterial infection, vitiligo, and psoriasis. Traditional Chinese medicine theory said that it acted on warming kidney and activating yang, promoting inspiration, and treating diarrhea [1, 2]. In addition, it is also used as healthy product and food supplement in our daily life. Phytochemical studies on* P. corylifolia* revealed that coumarins, flavonoids, and monoterpene phenols were its main active components [3–5]. Modern pharmacological and clinical studies showed that related compounds of* P. corylifolia* possessed a variety of biological activities, such as anticancer effect [6, 7], antioxidant activity [8], antimicrobial activity [9], inhibition of DNA polymerase [10], prevention of diabetes [11], and inhibition of papilloma formation. Particularly,* P. corylifolia* extract contained a number of flavonoids, and their bioactivities have attracted more attention [12]. For example, isobavachin has a potent oestrogenic effect and could regulate the body's endocrine disorders so as to achieve the effect of relieving menopausal syndrome [13]. Bavachin and corylin have been shown to stimulate osteoblastic proliferation in vivo, which might be a useful treatment for osteoporosis [14]. Neobavaisoflavone in combination with TRAIL had effect of inducing apoptosis in prostate cancer cells [15]. Isobavachin and isobavachalcone showed potent antioxidant activity in microsomes and inhibited oxygen consumption induced by lipid peroxidation [16]. All these results revealed that* P. corylifolia* may be a potentially promising drug entity which can be developed for treating human disease. Thus, developing a simple, sensitive, and reliable method to assess the quality of medicinal material is necessary.

In Chinese Pharmacopoeia, psoralen and isopsoralen were selected as markers for assessing the quality of the herb and some related preparations. However, this method could only determine a few of the marker compounds while ignoring other similarly important active ingredients in* P. corylifolia*, which was insufficient to provide the chemical information for quality assessment. A large number of studies showed that rich varieties of flavonoids were widely contained in this plant, including flavonoids, isoflavonoids, flavanones, and prenylated flavonoids. These natural flavonoids have been reported to possess plenty of biological activities. Therefore, a comprehensive method would be developed to evaluate content of flavonoids in* P. corylifolia* which allowed the determination of multiple constituents.

**Figure 1:**
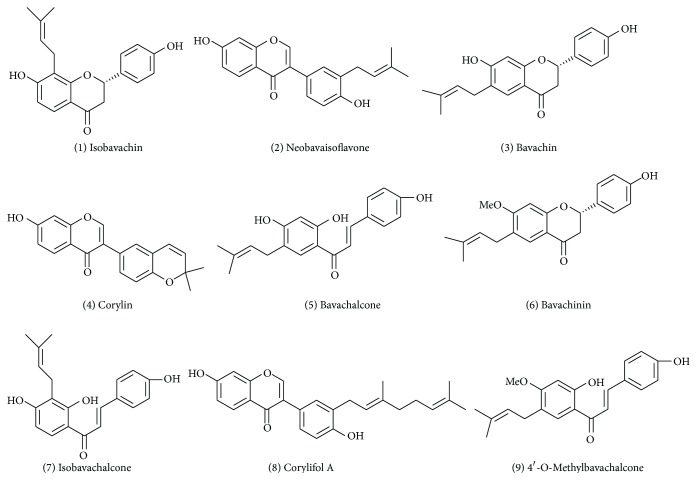
The chemical structures of the investigated compounds.

In this work, a simple high-performance liquid chromatography coupled with diode array detector (HPLC-DAD) method was established for determination of flavonoids (shown in Figure 1) from* P. corylifolia*. This method was successfully applied for identification and quantitation of 9 natural flavonoids, including isobavachin, neobavaisoflavone, bavachin, corylin, bavachalcone, bavachinin, isobavachalcone, corylifol A, and 4′-O-methylbavachalcone. In addition, neobavaisoflavone was chosen as the standard to develop a QAMS method, because it is easily available. Furthermore, the content of neobavaisoflavone was relatively stable in* P. corylifolia* [17]. The relative correction factors (RCFs) were calculated by a function formula for the quantitative analysis of the content of other flavonoids and then compared with the content of the external standard method to verify the accuracy of QAMS method. By QAMS method, the analytes (lacking reference standards for quantification) could be quantified with only small amounts of standards required to calculate the quantitative RCF. Currently, QAMS method has been applied for quality evaluation of a variety of Chinese herbal medicines, such as Phellodendri Chinensis Cortex, Astragali Radix,* Schisandra chinensis*,* Panax ginseng*, and* Panax notoginseng* [18–21]. In this sense, QAMS method may be a new way to make up for the lack of standards.

Previously, a number of studies have focused on identifying and characterizing of constituents in the fruits of* P. corylifolia* by using HPLC, MS spectra, gas chromatography (GC), and micellar electrokinetic capillary chromatography. However, these methods ignored the important active ingredient of flavonoids, which was insufficient to provide the information for quality evaluation of* P. corylifolia*. To the present, the method characters of flavonoids in* P. corylifolia* have never been reported. In this work, a QAMS method has been established for determination of a variety of flavonoids in* P. corylifolia* for the first time.

## 2. Experimental

### 2.1. Reagents and Materials

Acetonitrile (US TEDIA Reagent Company, HPLC grade), the water purified with a EPED water purification system from Nanjing EPED system (Nanjing, China), methanol (Nanjing Chemical Reagent Co., AR), and other reagents were of analytical grade. Standards of reference substance were purchased from Shanghai U-sea Biotech (Shanghai, China). The purity of all 9 marker constituents was more than 99%. The structures of compounds are shown in Figure 1.

### 2.2. Preparation of Standard Solutions

Stock solutions of individual standards at a concentration of 0.5 mg/mL were prepared by dissolving the compounds in methanol. Working solution of mixtures of all the standards was prepared immediately before analyses by diluting the stock solutions, to attain the required concentrations for calibration measurements. The stock and working solutions of standards were all prepared in dark brown volumetric flasks and stored at 4°C.

### 2.3. Preparation of the Sample Solution

13 batches of* P. corylifolia* from different regions were collected as shown in Table 1, and the authentication of samples was conducted by Professor Jianwei Chen of Nanjing University of Traditional Chinese Medicine. The seeds were pulverized and the powder was screened through 60-mesh sieve. And 0.1 g of the powder was extracted by refluxing with 50 mL of ethanol for 1 h. The extraction was cooled to room temperature and filtered, and then the filtrate was evaporated to dryness over water bath. The residue was completely transferred to a volumetric flask and diluted to exact 10 mL. The solution was filtered through a 0.45 *μ*m membrane filter before being injected to HPLC analysis. All sample solutions were stored at 4°C.

### 2.4. Instrument and Chromatographic Conditions

Chromatographic separation was achieved on Agilent 1100 high-performance liquid chromatography equipped with DAD detector (Agilent, USA) and Waters 2695 high-performance liquid chromatography equipped with 2998 detector (Waters, USA). The separation was carried out on a Purospher C18 column (150 mm × 4.6 mm i.d., 5 *μ*m) under the following chromatographic conditions: the injection volume was 10 *μ*L. The column temperature was maintained at 35°C. The wavelength of UV detection was set at 250 nm. The mobile phase was composed of acetonitrile (A) and water solution (B) with gradient elution system (0–10 min, 10%–45% A; 10–50 min, 45%–80% A; 50–55 min, 80%–90% A; 55–60 min, 90%–15% A) at a flow rate of 1.0 mL/min.

## 3. Results and Discussion

### 3.1. Method Validation

#### 3.1.1. Calibration Curves, Limits of Detection, and Limits of Quantity

The calibration curves were plotted with a series of concentrations of standard solutions. As showed in Table 2, good calibration curves of 9 compounds were obtained. High correlation coefficient values (*R* ^2^ > 0.9990) were showed with good linearity at a relatively wide range of concentrations. LOD and LOQ of 9 marker compounds were within range of 0.4780–2.805 *μ*g/mL and 1.872–9.349 *μ*g/mL, respectively, which showed a high sensitivity under the chromatographic condition we established.

#### 3.1.2. Precision

Method precision was checked by intraday and interday variability. The intraday variability was carried out by injection of the same standard solution six consecutive times in the same day. The interday variability was carried out for successive 2 days using the same solution. The RSD values obtained were summarized in Table 3. From the results, the developed method was found to be precise with intraday variability RSD values between 0.16% and 1.4% and interday variability RSD values between 0.82% and 2.8%.

#### 3.1.3. Stability, Repeatability, and Recovery

The stability of the sample solutions was analyzed at 0, 2, 4, 8, 12, and 24 h at room temperature. It was found that the sample solutions were stable within 24 h (RSD ≤ 1.8%). To confirm the repeatability of the method, six independently prepared solutions from the same sample (S7) were analyzed. The RSD values of the peak area were 0.56%–2.1%, respectively. The recovery was performed by adding a known amount of individual standards into a certain amount (0.50 g) of* Psoralea* sample (S7). The mixture was extracted and analyzed by using the method mentioned above. Six replicates were performed for the determination. The recoveries of the 9 compounds which were shown in Table 3 ranged from 94.94% to 103.5% with RSD ≤ 2.6%.

### 3.2. Application to Sample Analyses

**Figure 2:**
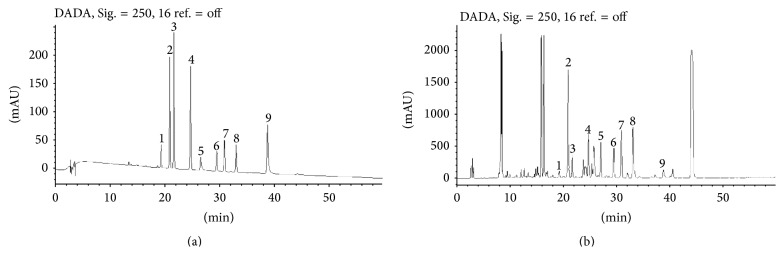
A chromatogram of mixture of standard compounds (a); a chromatogram of* P. corylifolia* L. from Jiangsu Province (b). Peak 1: isobavachin, peak 2: neobavaisoflavone, peak 3: bavachin, peak 4: corylin, peak 5: bavachalcone, peak 6: bavachinin, peak 7: isobavachalcone, peak 8: corylifol A, and peak 9: 4′-O-methylbavachalcone.

The method was subsequently applied to simultaneous quantitative analysis of 9 components in thirteen batches of* P. corylifolia*. The chromatograms of mixture of standard compounds and* P. corylifolia* L. from Jiangsu Province were shown in Figure 2. The contents of the 9 components were exhibited in Table 6.

### 3.3. Quantitative Analysis of Multicomponents by Single Marker

Neobavaisoflavone was chosen as a standard for the quantitative analysis of other flavonoids, including isobavachin, bavachin, corylin, bavachinin, isobavachalcone, corylifol A, and 4′-O-methylbavachalcone, for it extensively exists in various* P. corylifolia* L. and is commercially available (in order to eliminate interference due to the unsatisfied separation and low contents of bavachalcone and corylin, the RCF of these components was not calculated). The RCF is a constant of proportionality in a computational formula and can be calculated as follows:

(1) $$\begin{array}{c} f_{km}=\frac{f_{k}}{f_{m}}=\frac{\left(W_{k}\times A_{m}\right)}{\left(W_{m}\times A_{k}\right)}, \end{array}$$

where *A*_*k*_ is the peak area of the standard solution, *A*_*m*_ is the peak area of analyte, *W*_*k*_ is the concentration of standard solution, and *W*_*m*_ is the concentration of analyte. The value of *f* was calculated under different injection volumes and the average value (*f*) was used to calculate the content of analyte according to the formula. The values of *f* under different injection volumes (1, 2, 4, 6, 8, 10, 16, and 20 *μ*L) were showed in Table 4. The values of *f* with different columns and instruments were displayed in Table 5. Comparison with the results of absolute quantitative determinations and the accuracies were listed in Table 7.

In order to obtain a good separation, it is critical to select a suitable mobile phase, elution mode, and detection wavelength. In the present study, different mobile phases were investigated with gradient elution, including acetonitrile-water and methanol-water with modifiers such as acetic acid, formic acid, and phosphoric acid at different concentrations. The mobile phases were investigated in chromatographic peak shapes and signal-to-noise ratios (*S*/*N*) of the analytics. The detection wavelength was selected according to the maximum absorption wavelengths of mixed standard solutions. Under the chromatographic conditions as described previously, all the 9 components could be baseline separated within 60 min. Since no significant differences were observed on *S*/*N* and peak shape with different mobile phases, acetonitrile-water was chosen, which was the simplest combination to achieve reliable quantification of these components.

## 4. Conclusions

There were numerous methods reported for quality control of* P. corylifolia*, which were only focused on the contents of few ingredients. However, these methods ignored active ingredients of flavonoids. In this paper, an HPLC method for simultaneous determination of major components in* P. corylifolia* offered a powerful and rational way to guarantee the quality of this herb.

Based on this, a QAMS method was developed for determining the contents of other flavonoids. In the case of lacking reference substances, QAMS method still could be used to complete quantitative analysis of multiple ingredients, which provided basis for comprehensive evaluation of quality of the herb. Finally, compared with the results of absolute quantitative determinations, the accuracies of QAMS were between 92.89% and 109.5%. The RSD values of *f* in different injection volumes were between 2.3% and 3.6%. The results obtained from QAMS suggested that it was a convenient and accurate method to determine multicomponents when some authentic standard substances were unavailable. It can be used to quantify and control the quality of* P. corylifolia*. The QAMS method may be a new strategy to break through the choke point of lacking standard substances in phytochemical analysis. However, the feasibility of application of QAMS for determination of different types of components is still to be investigated.

## Supplementary Material

The seeds of *Psoralea corylifolia L.* (Fabaceae) are a commonly used medicinal herb in eastern Asia with many beneficial effects in clinical therapies. In this work, a simple high-performance liquid chromatography coupled with diode-array detector (HPLC-DAD) method for simultaneous determination of main 9 natural flavonoids of *Psoralea corylifolia L*.

## Figures and Tables

**Table 1:** The information of the crude pieces of *P. corylifolia* L.

Number	Origin	Batch	Manufacturer
S1	Guizhou	120521	Nanjing Haichang Chinese Medicine Pieces Factory
S2	Yunnan	1012928	Nanjing Haichang Chinese Medicine Pieces Factory
S3	Yunnan	120327	Nanjing Haichang Chinese Medicine Pieces Factory
S4	Zhejiang	110717	Nanjing Haichang Chinese Medicine Pieces Factory
S5	Guangxi	121108	Nanjing Haichang Chinese Medicine Pieces Factory
S6	Yunnan	121104	Shanghai Lei Yun Shang Chinese Medicine Pieces Factory
S7	Jiangsu	121001	Shanghai Lei Yun Shang Chinese Medicine Pieces Factory
S8	Henan	120524	Shanghai Lei Yun Shang Chinese Medicine Pieces Factory
S9	Henan	120521	Shanghai Lei Yun Shang Chinese Medicine Pieces Factory
S10	Burma	120728	Shanghai Tong Ji Tang Pharmaceutical Co., Ltd.
S11	Yunnan	120521	Shanghai Tong Ji Tang Pharmaceutical Co., Ltd.
S12	Yunnan	120524	Bozhou Yonggang Pieces Factory
S13	Jiangxi	121108	Bozhou Yonggang Pieces Factory

**Table 2:** Calibration curves for the 9 compounds determined.

Peak number	Analyte	Calibration curve	*R* ^2^	Linear range (*µ*g/mL)	LOD (*µ*g/mL)	LOQ (*µ*g/mL)
1	Isobavachin	*y* = 6094.2*x* − 47.492	0.9994	18.75–300.0	2.615	8.718
2	Neobavaisoflavone	*y* = 28072*x* − 228.09	0.9994	16.88–270.0	0.5610	1.872
3	Bavachin	*y* = 9536.0*x* − 72.258	0.9997	9.375–300.0	2.612	8.706
4	Corylin	*y* = 52057*x* − 175.41	0.9996	7.500–120.0	0.4780	1.594
5	Bavachalcone	*y* = 10586*x* − 84.429	0.9992	12.50–200.0	1.809	6.029
6	Bavachinin	*y* = 8039.5*x* − 89.087	0.9996	28.13–450.9	2.805	9.349
7	Isobavachalcone	*y* = 9564.5*x* − 112.19	0.9995	22.50–360.0	2.704	9.012
8	Corylifol A	*y* = 19363*x* − 148.88	0.9996	12.50–200.0	1.014	3.381
9	4′-O-Methylbavachalcone	*y* = 13683*x* + 0.87600	0.9997	7.656–245.0	1.853	6.178

**Table 3:** Accuracy, repeatability, and recovery of markers in *P. corylifolia* L. samples (*n* = 6).

Peak number	Analyte	Intraday RSD (%)	Interday RSD (%)	Repeatability RSD (%)	Recovery (%)
1	Isobavachin	0.33	1.6	1.8	97.23
2	Neobavaisoflavone	0.18	2.6	1.0	96.43
3	Bavachin	0.20	0.82	1.3	95.61
4	Corylin	0.16	1.3	1.0	103.5
5	Bavachalcone	1.3	2.8	1.1	100.4
6	Bavachinin	1.4	2.5	1.1	97.79
7	Isobavachalcone	0.30	2.3	0.56	94.94
8	Corylifol A	1.4	2.5	0.64	95.52
9	4′-O-Methylbavachalcone	1.1	2.2	2.1	98.45

**Table 4:** The values of *f* calculated under different injection volumes (*n* = 3).

Injection volume	Value of RCFs					
/*μ*L	*f*_1_	*f*_2_	*f*_3_	*f*_4_	*f*_5_	*f*_6_
1	0.712	3.03	0.859	1.94	0.734	1.38
2	0.702	2.95	0.852	1.94	0.745	1.42
4	0.699	2.95	0.849	1.92	0.745	1.34
6	0.687	2.91	0.833	2.00	0.776	1.43
10	0.672	2.89	0.827	2.00	0.774	1.46
16	0.651	2.80	0.803	2.01	0.780	1.47
20	0.656	2.86	0.806	2.04	0.786	1.48
Mean	0.683	2.92	0.833	1.98	0.763	1.43
RSD%	3.5	2.3	2.6	2.3	2.7	3.6

*f*_1_: *f*_isobavachin/neobavaisoflavone_; *f*_2_: *f*_bavachin/neobavaisoflavone_;

*f*_3_: *f*_bavachinin/neobavaisoflavone_; *f*_4_: *f*_isobavachalcone/neobavaisoflavone_;

*f*_5_: *f*_corylifol A/neobavaisoflavone_; *f*_6_: *f*_4'-O-methylbavachalcone/neobavaisoflavone_.

**Table 5:** The values of *f* calculated with different columns and instruments (*n* = 3).

Instrument	Column	Value of RCFs					
		*f*_1_	*f*_2_	*f*_3_	*f*_4_	*f*_5_	*f*_6_
Agilent 1100	Purospher C18	0.683	2.916	0.833	1.98	0.763	1.46
Agilent 1100	Alltima C18	0.676	2.928	0.814	2.07	0.778	1.52
Waters 2695	Purospher C18	0.672	2.956	0.799	2.02	0.774	1.44
Waters 2695	Alltima C18	0.688	2.935	0.803	2.12	0.780	1.48

**Table 6:** Results of absolute quantitative determination of marker compounds.

Number	Content (mg/g)								
	1	2	3	4	5	6	7	8	9
S1	—	9.124	4.367	0.7310	2.478	8.251	8.013	6.616	—
S2	—	9.647	5.043	0.7800	2.368	12.18	11.61	7.749	—
S3	—	9.538	5.280	1.031	2.983	9.386	11.17	7.432	1.049
S4	6.847	9.677	5.623	1.610	4.040	9.178	12.605	6.997	1.818
S5	8.115	9.058	3.503	1.187	3.328	6.416	8.731	7.237	2.004
S6	4.846	8.202	5.570	2.011	6.741	7.707	9.729	6.624	2.582
S7	8.915	7.837	4.075	1.478	3.744	7.547	7.948	5.962	2.024
S8	8.411	14.66	7.026	1.452	4.260	10.94	13.68	9.726	5.393
S9	—	10.29	4.517	0.6540	2.122	10.70	12.43	7.185	2.120
S10	—	5.413	3.437	1.349	2.928	4.229	7.519	4.892	1.466
S11	—	7.326	3.149	0.694	3.958	9.948	8.287	5.792	2.981
S12	—	5.474	3.145	0.638	1.768	6.040	6.964	3.437	1.208
S13	—	8.281	3.142	1.231	2.921	6.367	7.120	7.247	1.479

**Table 7:** Results of QAMS quantitative determination of marker compounds.

Number	1		3		6		7		8		9	
Sample	QAMS accuracy		QAMS accuracy		QAMS accuracy		QAMS accuracy		QAMS accuracy		QAMS accuracy	
	(mg/g)	(%)	(mg/g)	(%)	(mg/g)	(%)	(mg/g)	(%)	(mg/g)	(%)	(mg/g)	(%)
S1	—	—	9.393	102.9	4.229	96.84	8.092	99.86	6.750	102.0	—	—
S2	—	—	9.476	98.23	4.773	94.65	11.52	99.33	7.650	98.73	—	—
S3	—	—	9.665	101.3	5.195	98.39	11.41	102.1	7.541	101.5	1.064	101.5
S4	6.755	98.66	9.846	101.7	5.607	99.72	13.07	103.7	7.070	101.0	1.853	102.0
S5	8.682	107.0	9.740	107.5	3.383	96.57	9.231	105.7	7.809	107.9	2.168	108.2
S6	4.660	96.15	8.448	103.0	5.705	102.4	10.09	103.7	6.839	103.2	2.714	105.1
S7	9.329	104.6	8.045	102.7	3.349	96.92	8.028	101.0	6.080	102.0	2.124	105.0
S8	8.300	98.69	15.03	102.5	7.057	100.4	13.97	102.1	9.932	102.1	5.386	99.87
S9	—	—	10.31	100.2	4.252	94.13	12.61	101.5	7.136	99.33	2.116	99.82
S10	—	—	5.152	94.88	3.720	108.2	7.773	103.4	5.030	97.26	1.495	102.0
S11	—	—	7.159	97.73	2.925	92.89	8.077	97.46	5.640	97.38	3.015	101.1
S12	—	—	5.597	102.2	2.985	94.92	7.200	103.4	3.284	95.57	1.323	109.5
S13	—	—	9.052	109.3	3.016	96.00	7.309	102.6	7.852	108.35	1.609	108.8

## Abbreviations

QAMS: Quantitative analysis of multicomponents by single marker
RCF: The relative correction factor
HPLC-DAD: High-performance liquid chromatography coupled with diode array detection.
