# Supplementary figures and images for: Development of an HPLC Method for Absolute Quantification and QAMS of Flavonoids Components in Psoralea corylifolia L
Source: J Anal Methods Chem. 2015 Oct 26;2015:792637. doi: 10.1155/2015/792637 (PMC4637493; doi:10.1155/2015/792637)

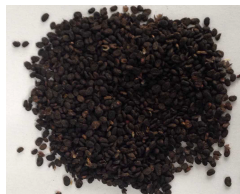

*Psoralea corylifolia* L.

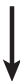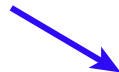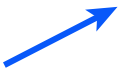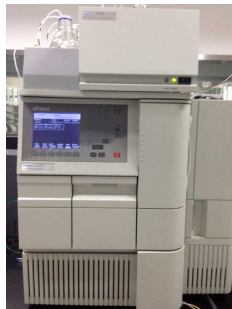

**QAMS**

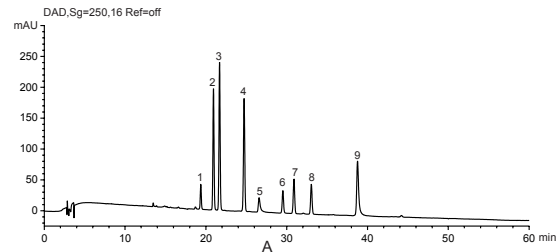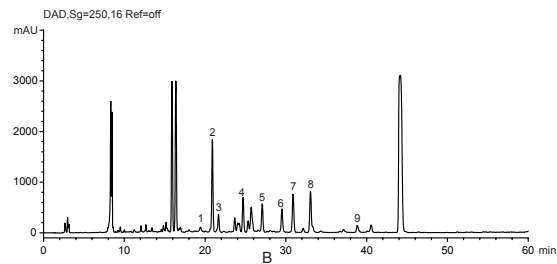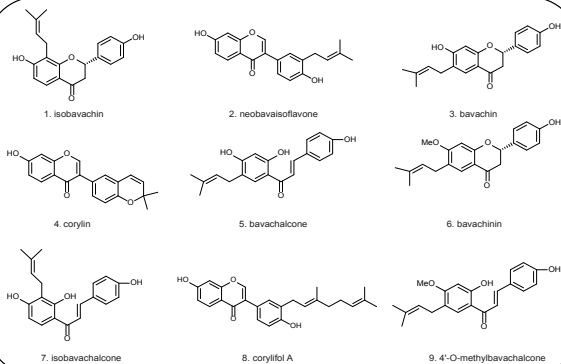

Supplement: Supplementary file 1 — The seeds of Psoralea corylifolia L. (Fabaceae) are a commonly used medicinal herb in eastern Asia with many beneficial effects in clinical therapies. In this work, a simple high-performance liquid chromatography coupled with diode-array detector (HPLC-DAD) method for simultaneous determination of main 9 natural flavonoids of Psoralea corylifolia L. [file 792637.f1.pdf]
